# A pilot study of stereotactic body radiation therapy (SBRT) after surgery for stage III non-small cell lung cancer

**DOI:** 10.1186/s12885-018-5039-5

**Published:** 2018-11-29

**Authors:** Anurag K. Singh, Mark Hennon, Sung Jun Ma, Todd L. Demmy, Anthony Picone, Elizabeth U. Dexter, Chumy Nwogu, Kristopher Attwood, Wei Tan, Gregory M. Hermann, Simon Fung-Kee-Fung, Harish K. Malhotra, Sai Yendamuri, Jorge A. Gomez-Suescun

**Affiliations:** 1 Department of Radiation Medicine, Roswell Park Comprehensive Cancer Center, 665 Elm St, Buffalo, NY USA; 2 Department of Thoracic Surgery, Roswell Park Comprehensive Cancer Center, 665 Elm St, Buffalo, NY USA; 3 Department of Biostatistics, Roswell Park Comprehensive Cancer Center, 665 Elm St, Buffalo, NY USA

**Keywords:** Post-operative, Adjuvant, SABR, RT, Mediastinum

## Abstract

**Background:**

Standard therapy for stage III non-small cell lung cancer with chemotherapy and conventional radiation has suboptimal outcomes. We hypothesized that a combination of surgery followed by stereotactic body radiation therapy (SBRT) would be a safe alternative.

**Methods:**

Patients with stage IIIA (multistation N2) or IIIB non-small cell lung cancer were enrolled from March 2013 to December 2015. The protocol included transcervical extended mediastinal lymphadenectomy (TEMLA) followed by surgical resection, 10 Gy SBRT directed to the involved mediastinum/hilar stations and/or positive surgical margins, and adjuvant systemic therapy. Patients not suitable for anatomic lung resection were treated with 30 Gy to the primary tumor. The primary efficacy end-point was the proportion of patients with grade 3 or higher adverse events (AE) or toxicities.

**Results:**

Of 10 patients, 7 patients underwent neoadjuvant chemotherapy. All patients had TEMLA. Nine of 10 patients underwent surgical resection. The remaining patient had an unresectable tumor and received 30 Gy SBRT to the primary lesion. All patients had post-operative SBRT. Median follow-up was 18 months. There were no perioperative mortalities. Six patients had any grade 3 AEs with no grade 4–5 AEs. Of these, 4 were not attributable to radiation. Pulmonary-related grade 3 AEs were experienced by 2 patients. There were no failures within the 10 Gy volume. Overall survival and progression-free survival rates at 2 years were 68% (90% CI 36–86) and 40% (90% CI 16–63), respectively.

**Conclusions:**

In carefully selected patients with locally advanced non-small cell lung cancer, combining surgery with SBRT was well tolerated with no local failure.

**Trial registration:**

ClinicalTrials.gov identifying number NCT01781741. Registered February 1, 2013.

## Background

The standard of care for unresectable stage III non-small cell lung cancer (NSCLC) remains concurrent chemoradiation [1, 2]. Even among patients with excellent performance status and limited disease-related weight loss prior to their treatments, the 5-year overall survival remains 16% with significant toxicity [1, 2]. Radiation dose escalation worsened overall survival with no improvements in local control of less than 70% [3]. Surgery is generally reserved for very highly selected patients, such as those with persistent N2 disease following neoadjuvant chemotherapy or chemoradiation [4, 5].

When compared to conventionally fractionated radiation therapy as used in chemoradiation, stereotactic body radiation therapy (SBRT) has equivalent survival and local control with less toxicity, improved quality of life, and shortened duration of treatment in patients with early stage NSCLC [6]. In addition, incidental radiation to mediastinal lymph nodes can be substantial by SBRT and may be potentially therapeutic for low-volume, subclinical nodal diseases after nodal resection [7]. Kepka et al. described a dose response for reduced nodal recurrence with an incidental radiation threshold of approximately 15Gy delivered in non-SBRT doses (1.2–4.0 Gray (Gy) per fraction), reflecting a BED_10_ ranging from 16.8 to 21Gy. Thus, the SBRT dose of 10 Gy delivered in 1 fraction (BED_10_ = 20Gy) to the mediastinum/hilum was chosen for investigation [8]. Recently, it has been reported that 10Gy in 1 fraction of thoracic radiation can improve quality of life and is well tolerated in patients undergoing palliative chemotherapy for NSCLC [9, 10]. Our aim for this study was to evaluate 10Gy in 1 fraction for definitive post-operative treatment. Additionally, a novel method for mediastinal nodal resection is transcervical extended mediastinal lymphadenectomy (TEMLA). It has been well tolerated with high sensitivity and specificity for nodal staging [11].

Currently, the use of SBRT in stage III NSCLC has been limited to delivering an additional boost to the residual primary tumor mass [12]. SBRT to the central thorax is associated with significant toxicity [13]. However, we hypothesized that the combination of TEMLA to debulk the disease followed by a relatively low dose of single fraction SBRT (10 Gy)) would be a safe alternative to conventionally fractionated radiation therapy and that it may potentially yield improved local control and toxicity profiles. This report details a pilot, single-center study to assess outcomes of TEMLA followed by SBRT for stage III NSCLC.

## Methods

### Patient eligibility

This was an institutional review board approved prospective clinical trial (NCT01781741). Eligibility criteria included: histologically confirmed stage III/IV NSCLC, age 18 or older, and Eastern Cooperative Oncology Group (ECOG) Performance Status of 2 or less. Patients with stage IV NSCLC were eligible if limited to solitary, limited volume metastasis in brain, bone, or adrenal glands. All patients underwent complete staging with PET-CT and brain MRI. Neoadjuvant chemotherapy was allowed at the discretion of medical oncologist. Patients with open thoracotomy as part of their surgical treatments were ineligible, as this group was considered too high risk for a pilot study evaluating toxicity as an endpoint. Patients with known N2 disease or oligometastases prior to TEMLA were required to consent prior to TEMLA, whereas those found to have N2 disease during TEMLA were allowed to consent after TEMLA. Patients were not required to have known N2 disease prior to TEMLA. There was no restriction on the number of involved nodal stations or bulk of disease. All patients underwent pulmonary function tests. No specific pulmonary function test requirement was utilized in this protocol, although our institutional practice is to consider curative-intent surgical resection in only those patients with forced expiratory volume in 1 second (FEV1) and/or diffusing capacity of lung for carbon monoxide (DLCO) > 50% predicted value. Surgical and medical candidacy for surgical resection was determined at the discretion of the thoracic surgeon. Surgical and medical candidacy for surgical resection was determined at the discretion of the thoracic surgeon. Contralateral mediastinal involvement was not an absolute contraindication.

### Treatment

Formal long term adverse event assessment beyond 12 weeks was not done (to avoid counting of toxicities with standard adjuvant chemotherapy) and is a limitation of this study, therefore, we are unable to report on any late toxicity secondary to RT. All patients received TEMLA to remove their mediastinal nodes. The primary tumor was treated with video assisted thoracoscopic surgery (VATS) or SBRT to 30 Gy in a single fraction if inoperable by VATS. Any minimally invasive procedure with VATS (lobectomy, segmentectomy, wedge resection) was allowed. Evaluation of hilar nodes was completed with either TEMLA or VATS. SBRT of 10 Gy was given to any positive mediastinal or hilar lymph node region or positive surgical margins [7]. TEMLA and SBRT were required to be completed in the first 2 months. Adjuvant chemotherapy was provided at any time 2 weeks after SBRT, at the discretion of the medical oncologist.

### TEMLA

The operative techniques of TEMLA at our institution were described previously [14–16]. Briefly, the procedure includes a 5 to 8 cm collar incision in the neck, sternal manubrium elevation, and the bilateral visualization of the vagus and laryngeal recurrent nerves, and the dissection of all mediastinal nodal stations except for the pulmonary ligament lymph nodes. In this study, video-assisted mediastinal lymphadenectomy (VAMLA) was allowed at the surgeon’s discretion.

### SBRT

The delivery method of SBRT at our center previously was reported [17]. Briefly, all patients were stabilized by Body Fix (Elektra, Stockholm, Sweden) immobilizer for CT simulation, and their respiratory motion was evaluated using Real-Time Position Management (Varian Medical System, Palo Alto, CA).

**Fig. 1:**
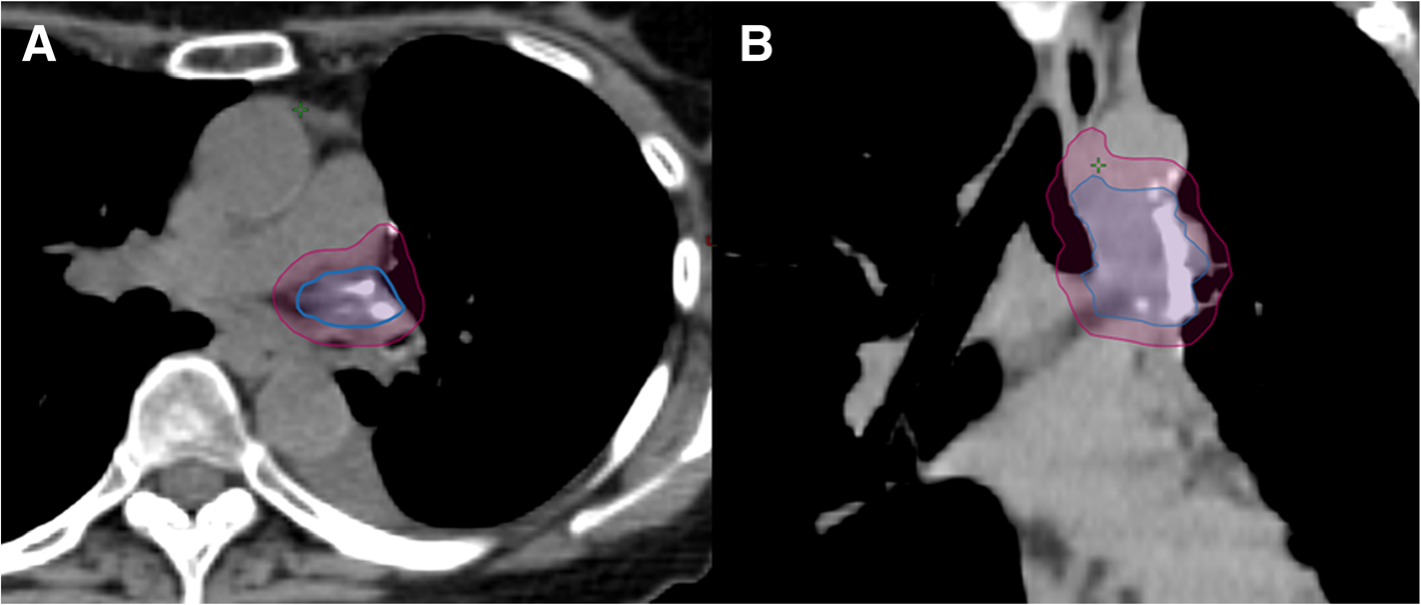
Example of the nodal contour for patient No. 3 who had a clinically positive level 10 lymph node and a positive level 4 lymph node from TEMLA. **a** Axial view **b**. Coronal view. Clinical Target Volume (CTV) (blue) and Planning Target Volume (PTV) (purple)

Treatment planning and normal tissue constraints were per RTOG 0915 for single fraction SBRT [18]. Either volumetric-modulated arc therapy (VMAT) or intensity-modulated radiation therapy (IMRT) were used with heterogeneity corrections. Clinical target volume (CTV) of the involved lymph nodes was defined by staging nodal evaluation including endobronchial ultrasound, PET/CT and pathologic findings at time of TEMLA. The CTV did not include any elective nodal station (e.g. in the case of a positive hilar nodal station, only that station was included in the CTV). An internal target volume (ITV) was generated using 4D CT or deep-inspiratory breath hold, and the planning target volume (PTV) was generated by adding a uniform 0.5 cm margin to the ITV. PTV of lymph nodes was drawn based on anatomic landmarks as described previously [7, 19]. Figure 1 provides an example of the CTV (blue) and PTV (purple) for a patient with a clinically positive level 10 lymph node and a positive level 4 L lymph node from TEMLA, covering only those respective lymph node regions.

The requirement was for 95% of PTV to be conformally covered by the prescription isodose surface, 30 Gy for the primary tumor site and 10 Gy for lymph nodes or positive surgical margin. Also, 99% of the PTV was required to receive a minimum of 90% of the prescription dose. The dose for all patients was prescribed to the prescription line at the edge of the PTV.

### Initial evaluation and follow-up

SBRT was delivered at least 2 weeks after any surgery and at most 8 weeks after TEMLA. Patients were followed at 6, 9, 12 months after SBRT and then every 6 months for the next 2 years. Imaging with either PET-CT or CT scan of the chest was completed at a minimum 12 weeks, 1 year, 3 years, and 5 years post-SBRT or if clinically indicated. Adverse events were assessed until 12 weeks after SBRT.

### Outcome assessments

The primary endpoint was the frequency and relative frequency of grade 3 or higher adverse events (AE) or toxicities. Toxicities were evaluated based on Common Terminology Criteria for Adverse Events (CTCAE) version 4.0. They were estimated using a 90% confidence interval obtained by the Clopper-Pearson method. Secondary endpoints included: median duration of time between SBRT and chemotherapy, and between TEMLA and SBRT, overall survival (OS) and progression-free survival (PFS) and quality of life (QOL) scores. Time from SBRT to chemotherapy was estimated using a 90% confidence interval obtained by standard Kaplan-Meier methods. OS and PFS were evaluated by the Kaplan-Meier method. The median survival, 1- and 2-year survival rates were estimated with 90% confidence intervals calculated from the time of TEMLA. QOL scores were obtained by the EORTC QLQ-C30 and EORTC QLQ Lung Cancer-Specific Module questionnaires, which were required to be administered at 6 and 12 week follow-up, and allowed to be administered at each additional follow-up. A linear transformation was used in order to standardize the raw QOL scores in a scale from 0 to 100 [20, 21]. Global scores were summarized by time-point using the mean, median, standard deviation, and range. The sign test was used in order to compare the QOL scores with the baseline scores. All tests were two-sided and tested at a 0.1 nominal significance level. Pulmonary function tests were summarized by visit using the mean, median, standard deviation, and range. SAS (version 9.4, Cary, NC) software was used for all statistical analyses.

## Results

Between March 2013 and December 2015, 16 patients were screened for study. Of these 16, seven patients received neoadjuvant chemotherapy, and all patients underwent TEMLA. Two progressed during neoadjuvant chemotherapy and 4 were node negative following chemotherapy at the time of TEMLA. The remaining 10 patients with stage IIIA or IIIB NSCLC were enrolled in the study. Although Stage IV patients and those with positive surgical margins were eligible, none were enrolled. Baseline characteristics, demographics, and outcomes are described in Table 1. The majority was former smokers and had ECOG performance status of 0. Nine patients underwent VATS surgical resection of the primary tumors, of those, 8 underwent lobectomy and 1 underwent a wedge resection. Four patients had clinical or pathologically confirmed N3 disease, all by virtue of contralateral mediastinal lymph node involvement. Three patients had subcarinal involvement.

**Table 1:** Patient, tumor, treatment characteristics, outcomes

Patient No.	Clinical TNM	Pathologic TNM	TEMLA Positive Stations (# positive/total)	PTV	Recurrence	Alive
1	T3 N2 M0	N2	4R(3/13)	53.35	N	N
2	T3 N2 M0	T3 N2	4R(11/14) 10R(1/4)	170.21	Y (SCV LN s/p RT, currently NED)	Y
3	T2aN2 M0	ypT2bN2	4 L(1/11)	47	Y (Brain s/p RT, currently NED)	Y
4	T1N3M0	ypT1bN3	4R(7/7) 5(1/1) 6(3/3) 7(1/1)	166	Y (liver)	N
5	T1aN3M0	T1aN3	4R(2/3)	52.2	N	Y
6	T2N3M0	T4 N3	4R(4/4) 4 L(1/1) 7(1/1) 10R(2/2) 11R(1/1)	154.75	Y (SCV LN s/p RT)	N
7	T2N3M0	ypT2aN2	5(2/2) 10 L(3/5)	89.19	Y (Brain s/p RT, currently NED)	Y
8	T1bN2 M0	ypT2aN2	7(2/9)	56.16	Y (pleural effusion)	N
9	T1aN2 M0	ypT1aN2	4 L(1/1) 11 L(3/8)	33.14	N	Y
10	T2aN2 M0	ypT1bN2	2R(2/2) 4R(5/6) 7(2/3) 9R(1/1) 10R(2/4) 11R(1/1)	94.2	Y (liver and brain; ALK-mutant)	Y

Patient 1 received SBRT to the primary tumor

All received 10 Gy to the positive nodal areas

All patients except 1,2 had adenocarcinoma

All patients except 2, 5, 6 received neoadjuvant chemotherapy

All patients except 3 received adjuvant chemotherapy

*M* male, *F* female, *PTV* = Planning Target Volume in cubic centimeters, *SCV* supraclavicular, *s/p* status post, *RT* radiation therapy, *NED* no evidence of disease

Patient 1 had a primary tumor that was deemed unresectable during the surgical exploration. The patient received 30 Gy in a single fraction to the primary tumor with concurrent 10 Gy in a single fraction to the mediastinal lymph nodes. A complete response was achieved by follow-up PET scan performed 4 months after SBRT; the patient had a good performance status at that time; however, the patient expired of unknown causes 3 months later.

All patients had positive mediastinal/hilar lymph node metastases and underwent post-operative SBRT in the mediastinal/hilar region. The median time from TEMLA to SBRT was 35.5 days (range 24–37). All patients received standard doublet adjuvant chemotherapy; the median time from SBRT to chemotherapy was 14 days (range 13–37).

With a minimum follow-up of 18 months, 6 patients are alive. No perioperative mortality was reported. As shown in Tables 2, 6 (60%) of patients reported grade 3 AEs (90% CI 30–85). In 4 patients these AEs were related to chemotherapy and not attributable to radiation. Two patients reported grade 3 pulmonary-related AEs (dyspnea and cough). No grade 4–5 AEs were observed.

**Table 2:** Adverse Events (AE)

Adverse Event		Grade		
		1	2	3
System Organ Class	Specific Term			
Cardiac disorders	Palpitations	1	0	0
Gastrointestinal disorders	Any AE - Maximum	3	2	0
	Dyspepsia	1	0	0
	Dysphagia	1	2	0
	Nausea	1	0	0
	Esophagitis	1	0	0
General disorders and administration site conditions	Any AE - Maximum	2	3	0
	Asthenia	0	1	0
	Fatigue	1	2	0
	Non-cardiac chest pain	1	0	0
	Sensation of foreign body	0	1	0
Respiratory, thoracic and mediastinal disorders	Any AE - Maximum	1	5	2
	Aspiration	0	1	0
	Atelectasis	0	1	0
	Cough	1	2	1
	Dyspnea	2	2	1
	Haemoptysis	1	0	0
	Pneumonitis	0	1	0
	Pneumonia	0	1	0
	Pneumothorax	0	1	0
	Productive cough	1	0	0
	Respiratory tract inflammation	1	0	0
Metabolism and nutrition disorders	Any AE - Maximum	4	2	0
	Decreased appetite	3	0	0
	Hypercalcemia	0	1	0
	Hyperkalemia	0	1	0
	Hypocalcaemia	2	0	0
	Hypomagnesaemia	0	1	0
Nervous system disorders	Dizziness	1	0	0
	Syncope	0	0	1
Musculoskeletal and connective tissue disorders	Chest Pain	0	1	0
Blood and lymphatic system disorders	Anemia	1	1	0
Metabolism and nutrition disorders	Any AE - Maximum Grade	2	1	0
Skin	Pruritis	0	1	0
	Discoloration	1	0	0
Investigations	Decreased Lymphocyte Count	0	0	2
	Blood creatine phosphokinase increased	0	0	1
Injury, poisoning and procedural complications	Incision Site Pain	0	1	0
ANY AE - Maximum Grade Seen		1	3	6

**Fig. 2:**
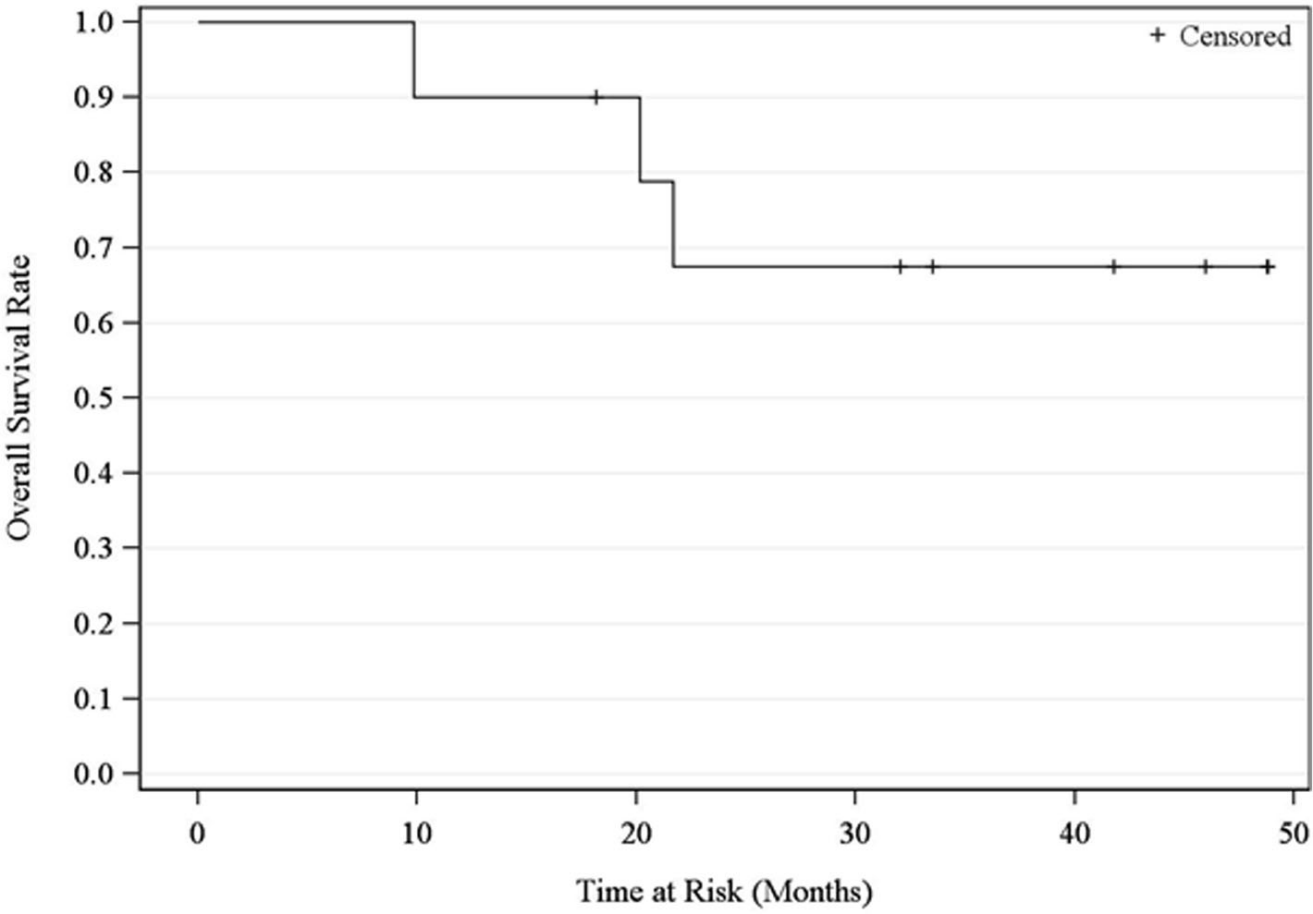
Overall Survival

**Fig. 3:**
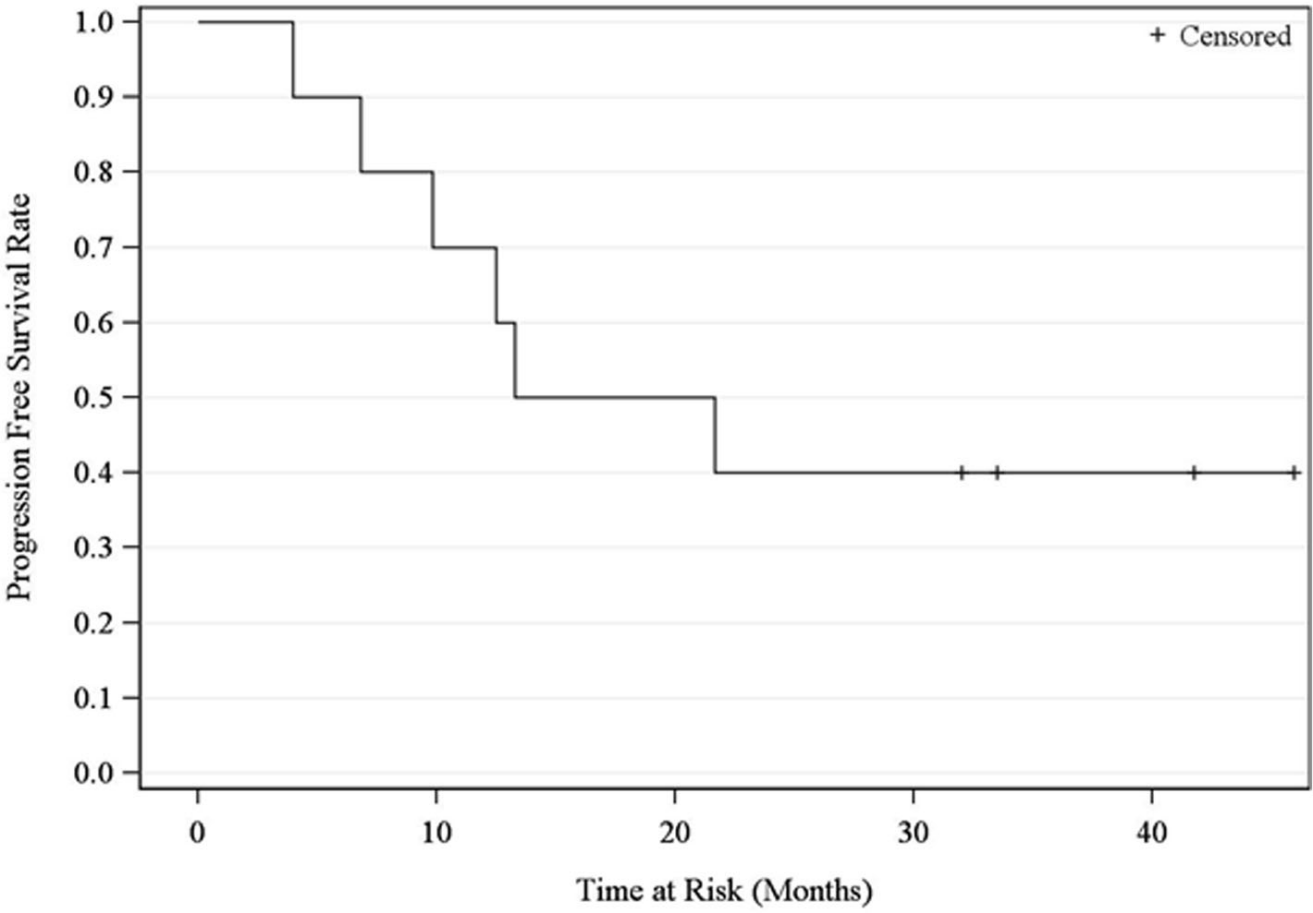
Progression Free Survival

OS and PFS rates at 2 years were 68% (90% CI 36–86) and 40% (90% CI 16–63), respectively (Figs. 2 and 3). Median OS has not yet been reached at 49 months and median PFS was 17.5 months. There was no failure observed within the 10 Gy volume. Also, as shown in Tables 1, 4 patients experienced distant failure and 3 experienced regional failure (2 supraclavicular and 1 pleural effusion).

Compared to the baseline quality of life scores, there were no statistically significant changes seen at any follow-up visit. Similarly, compared to the baseline, there appears to be no significant change seen in any measure of pulmonary function during follow-up.

## Discussion

This is the first report of a planned prospective study combining discretionary neoadjuvant chemotherapy, followed by limited surgery (VATS and TEMLA) followed by SBRT ultimately followed by adjuvant chemotherapy in locally advanced NSCLC. In patients with adequate pulmonary function and medically amenable to TEMLA/VATS with no distant disease, this combination of treatment modalities resulted in no local failures, limited toxicity, and the ability to proceed quickly to adjuvant chemotherapy.

Of the seven patients who completed neoadjuvant chemotherapy, all had persistent N2–3 disease after surgery. Patients with persistent N2+ disease were previously shown to have over 60% local-regional and distant failure rates at 5 years without postoperative radiation therapy and adjuvant chemotherapy [22, 23]. Several retrospective studies showed that the use of postoperative radiation therapy and additional chemotherapy following neoadjuvant chemotherapy and surgery in these patients reduced local-regional and distant failures, even improving overall survival in select patients [22, 24]. Our study is the first study to use postoperative SBRT, instead of conventionally fractionated radiation therapy, to shorten the time to the start of adjuvant chemotherapy.

Though systemic failure predominates, local failure remains a significant concern in locally advanced NSCLC treated with conventionally fractionated radiation and concurrent chemotherapy [25]. In this study, there were no local failures within the area treated by surgery and at least 10 Gy of SBRT.

In our study, 2 patients (20%) had supraclavicular nodal recurrences following TEMLA and SBRT. This pattern of failure is consistent with prior studies showing 2–16% of patients with supraclavicular nodal recurrences for NSCLC [26–28].

In this small study, 7 patients received neoadjuvant chemotherapy. Importantly, all patients tolerated surgery and SBRT well enough (only 2 patients with grade 3 toxicity possibly attributable to SBRT) to receive standard doublet adjuvant chemotherapy without significant delay (median time from TEMLA to SBRT was 35.5 days, SBRT to chemotherapy was 14 days). The overall G3+ toxicity rate of 60% compares favorably to a 76% rate reported with modern definitive chemoradiation treatment in the standard arm of RTOG 0617 [3]. Modern era post-operative radiation trials have demonstrated low rates of G3+ toxicity as low as 0%, although the concern for toxicity has long been established with a survival detriment identified by the PORT meta-analysis Trialist Group [29, 30]. We await the results of phase III modern post-operative trials such as the LungART to elucidate the future role of conventionally fractionated post-operative RT. Formal long term adverse event assessment beyond 12 weeks was not done (to avoid counting of toxicities with standard adjuvant chemotherapy) and is a limitation of this study; therefore, we are unable to report on any late toxicity secondary to RT.

OS and PFS rates at 2 years were 68 and 40% respectively. Despite the different radiation modality and sequence of adjuvant radiation and chemotherapy, the 1-year OS in our study was 90% which is consistent with the experience of 98% 1-year OS in a subgroup (*n* = 73) of patients on the ANITA trial who had pathological N2 disease who received adjuvant chemotherapy and RT [31]. The survival benefit of postoperative chemotherapy followed by conventional radiation for N2 diseases has further been shown in the secondary analysis of the ANITA trial (median survival 47.4 months vs 23.8 months) [32]. Such findings are consistent with other retrospective studies [33–35]. However, the receipt of adjuvant radiation early was shown to have survival benefits in a multicenter retrospective study, and the subsequent delay of receiving adjuvant chemotherapy was not associated with worse outcomes in prior studies. [36–38]. The ideal sequence of adjuvant regimens remains unclear, and the role of postoperative radiation will be elucidated in phase III LungART trial (NCT00410683).

Ultimately then, this study fulfilled our initial hypothesis that the combination of TEMLA to debulk the disease followed by a relatively low dose of single fraction SBRT (10 Gy) would be a safe alternative to conventionally fractionated radiation therapy, achieving excellent local control and toxicity profiles. Future studies of postoperative conventionally fractionated radiation versus SBRT should be considered.

In the interval, we have shown that single fraction SBRT prior to surgery is able to stimulate the immune system in patients with renal cell cancer [39]. Building upon this finding, our current study seeks to understand the immune effects of neoadjuvant single fraction SBRT in NSCLC patients (stage I to IIIA, NCT03348748) who will go on to surgery.

## Conclusion

For select patients with locally advanced NSCLC, the combination of neoadjuvant chemotherapy, surgery, and postoperative SBRT showed good local control with limited toxicity and a quicker transition to adjuvant chemotherapy. Such regimens warrant further study.

## Abbreviations

AE: Adverse events
CTCAE: Common Terminology Criteria for Adverse Events
CTV: Clinical target volume
ECOG: Eastern Cooperative Oncology Group
GTV: Gross tumor volume
Gy: Gray
IMRT: Intensity-modulated radiation therapy
ITV: Internal target volume
NSCLC: Non-small cell lung cancer
OS: Overall survival
PFS: Progression-free survival
PTV: Planning target volume
QOL: Quality of life
SBRT: Stereotactic body radiation therapy
TEMLA: Transcervical extended mediastinal lymphadenectomy
VAMLA: Video-assisted mediastinal lymphadenectomy
VATS: Video assisted thoracoscopic surgery
VMAT: Volumetric-modulated arc therapy

## References

[1] Furuse K, Fukuoka M, Kawahara M, Nishikawa H, Takada Y, Kudoh S (1999). Phase III study of concurrent versus sequential thoracic radiotherapy in combination with mitomycin, vindesine, and cisplatin in unresectable stage III non-small-cell lung cancer. J Clin Oncol.

[2] Curran WJ, Paulus R, Langer CJ, Komaki R, Lee JS, Hauser S (2011). Sequential vs. concurrent chemoradiation for stage III non-small cell lung cancer: randomized phase III trial RTOG 9410. J Natl Cancer Inst.

[3] Bradley JD, Paulus R, Komaki R, Masters G, Blumenschein G, Schild S (2015). Standard-dose versus high-dose conformal radiotherapy with concurrent and consolidation carboplatin plus paclitaxel with or without cetuximab for patients with stage IIIA or IIIB non-small-cell lung cancer (RTOG 0617): a randomised, two-by-two factorial phase 3 study. Lancet Oncol.

[4] Higgins KA, Chino JP, Ready N, Onaitis MW, Berry MF, D'Amico TA (2011). Persistent N2 disease after neoadjuvant chemotherapy for non-small-cell lung cancer. J Thorac Cardiovasc Surg.

[5] Meacci E, Cesario A, Cusumano G, Lococo F, D'Angelillo R, Dall'armi V (2011). Surgery for patients with persistent pathological N2 IIIA stage in non-small-cell lung cancer after induction radio-chemotherapy: the microscopic seed of doubt. Eur J Cardiothorac Surg.

[6] Nyman J, Hallqvist A, Lund JA, Brustugun OT, Bergman B, Bergstrom P (2016). SPACE - a randomized study of SBRT vs conventional fractionated radiotherapy in medically inoperable stage I NSCLC. Radiother Oncol.

[7] Martin KL, Gomez J, Nazareth DP, Warren GW, Singh AK (2012). Quantification of incidental mediastinal and hilar irradiation delivered during definitive stereotactic body radiation therapy for peripheral non-small cell lung cancer. Med Dosim.

[8] Kepka L, Maciejewski B, Withers RH (2009). Does incidental irradiation with doses below 50 gy effectively reduce isolated nodal failures in non-small-cell lung cancer: dose-response relationship. Int J Radiat Oncol Biol Phys.

[9] Rathod S, Jeremic B, Fidarova E, Faheem M, Lau FN, Sharma V, Ammar C, Forbe A, Agarwal JP (2017). Quality of Life Outcomes in a Phase 3 Randomized Trial of Optimization of Treatment of Advanced Non–small Cell Lung Cancer Using Radiation Therapy and Chemotherapy: IAEA Multicentric Randomized Phase 3 Study (NCT00864331). Int J Radiat Oncol Biol Phys.

[10] Jeremic B, Ghosh S, Fidavora E, Faheem M, Agarwal JP, Lau FN, Sharma V, Ammar C, Azmy A, Forbe A, Brincat S (2017). The International Atomic Energy Agency Randomized Trial on Chemotherapy With or Without Radiation Therapy in Advanced Non–small Cell Lung Cancer (NCT00864331). Int J Radiat Oncol Biol Phys.

[11] Zielinski M (2007). Transcervical extended mediastinal lymphadenectomy: results of staging in two hundred fifty-six patients with non-small cell lung cancer. J Thorac Oncol.

[12] Feddock J, Arnold SM, Shelton BJ, Sinha P, Conrad G, Chen L (2013). Stereotactic body radiation therapy can be used safely to boost residual disease in locally advanced non-small cell lung cancer: a prospective study. Int J Radiat Oncol Biol Phys.

[13] Bezjak A, Paulus R, Gaspar L, Timmerman R, Straube W, Ryan WF (2016). Efficacy and toxicity analysis of NRG oncology/RTOG 0813 trial of stereotactic body radiation therapy (SBRT) for centrally located non-small cell lung cancer (NSCLC). Int J Radiat Oncol Biol Phys.

[14] Huang M, Manuballa S, Demmy T, Yendamuri S (2013). Transcervical extended mediastinal lymphadenectomy - indications and technique. Indian J Surg Oncol.

[15] Kuzdzal J, Zielinski M, Papla B, Szlubowski A, Hauer L, Nabialek T (2005). Transcervical extended mediastinal lymphadenectomy--the new operative technique and early results in lung cancer staging. Eur J Cardiothorac Surg.

[16] Yendamuri S, Battoo A, Dy G, Chen H, Gomez J, Singh AK (2017). Transcervical extended Mediastinal lymphadenectomy: experience from a north American Cancer center. Ann Thorac Surg.

[17] Ma SJ, Serra LM, Syed YA, Hermann GM, Gomez-Suescun JA, Singh AK (2018). Comparison of single- and three-fraction schedules of stereotactic body radiation therapy for peripheral early-stage non-small-cell lung Cancer. Clin Lung Cancer.

[18] Videtic GM, Hu C, Singh AK, Chang JY, Parker W, Olivier KR (2015). A randomized phase 2 study comparing 2 stereotactic body radiation therapy schedules for medically inoperable patients with stage I peripheral non-small cell lung Cancer: NRG oncology RTOG 0915 (NCCTG N0927). Int J Radiat Oncol Biol Phys.

[19] Chapet O, Kong FM, Quint LE, Chang AC, Ten Haken RK, Eisbruch A (2005). CT-based definition of thoracic lymph node stations: an atlas from the University of Michigan. Int J Radiat Oncol Biol Phys.

[20] Fayers PM, Aaronson NK, Bjordal K, Groenvold M, Curran D, Bottomley A (2001). The EORTC QLQ-C30 scoring manual (3rd edition).

[21] Aaronson NK, Ahmedzai S, Bergman B, Bullinger M, Cull A, Duez NJ (1993). The European Organization for Research and Treatment of Cancer QLQ-C30: a quality-of-life instrument for use in international clinical trials in oncology. J Natl Cancer Inst.

[22] Taylor NA, Liao ZX, Stevens C, Walsh G, Roth J, Putnam J (2003). Postoperative radiotherapy increases locoregional control of patients with stage IIIA non-small-cell lung cancer treated with induction chemotherapy followed by surgery. Int J Radiat Oncol Biol Phys.

[23] Betticher DC, Hsu Schmitz SF, Totsch M, Hansen E, Joss C, von Briel C (2006). Prognostic factors affecting long-term outcomes in patients with resected stage IIIA pN2 non-small-cell lung cancer: 5-year follow-up of a phase II study. Br J Cancer.

[24] Amini A, Correa AM, Komaki R, Chang JY, Tsao AS, Roth JA (2012). The role of consolidation therapy for stage III non-small cell lung cancer with persistent N2 disease after induction chemotherapy. Ann Thorac Surg.

[25] Albain KS, Swann RS, Rusch VW, Turrisi AT, Shepherd FA, Smith C (2009). Radiotherapy plus chemotherapy with or without surgical resection for stage III non-small-cell lung cancer: a phase III randomised controlled trial. Lancet.

[26] Sulman EP, Komaki R, Klopp AH, Cox JD, Chang JY (2009). Exclusion of elective nodal irradiation is associated with minimal elective nodal failure in non-small cell lung cancer. Radiat Oncol.

[27] Kim E, Song C, Kim MY, Kim JS (2017). Long-term outcomes after salvage radiotherapy for postoperative locoregionally recurrent non-small-cell lung cancer. Radiat Oncol J.

[28] Garg S, Gielda BT, Turian JV, Liptay M, Warren WH, Bonomi P (2013). Patterns of regional failure in stage III non-small cell lung cancer treated with neoadjuvant chemoradiation therapy and resection. Pract Radiat Oncol.

[29] Boyer MJ, Gu L, Wang X, Kelsey CR, Yoo DS, Onaitis MW (2016). Toxicity of definitive and post-operative radiation following ipilimumab in non-small cell lung cancer. Lung Cancer.

[30] PORT Meta-analyis Trialists Group. Postoperative radiotherapy in non-small-cell lung cancer: systematic review and meta-analysis of individual patient data from nine randomised controlled trials. PORT Meta-analysis Trialists Group. Lancet. 1998;352(9124):257–63. 9690404

[31] Douillard JY, Rosell R, De Lena M, Carpagnano F, Ramlau R, Gonzales-Larriba JL (2006). Adjuvant vinorelbine plus cisplatin versus observation in patients with completely resected stage IB-IIIA non-small-cell lung cancer (adjuvant Navelbine international Trialist association [ANITA]): a randomised controlled trial. Lancet Oncol.

[32] Douillard JY, Rosell R, De Lena M, Riggi M, Hurteloup P, Mahe MA (2008). Impact of postoperative radiation therapy on survival in patients with complete resection and stage I, II, or IIIA non-small-cell lung cancer treated with adjuvant chemotherapy: the adjuvant Navelbine international Trialist association (ANITA) randomized trial. Int J Radiat Oncol Biol Phys.

[33] Robinson CG, Patel AP, Bradley JD, DeWees T, Waqar SN, Morgensztern D (2015). Postoperative radiotherapy for pathologic N2 non-small-cell lung cancer treated with adjuvant chemotherapy: a review of the National Cancer Data Base. J Clin Oncol.

[34] Mikell JL, Gillespie TW, Hall WA, Nickleach DC, Liu Y, Lipscomb J (2015). Postoperative radiotherapy is associated with better survival in non-small cell lung cancer with involved N2 lymph nodes: results of an analysis of the National Cancer Data Base. J Thorac Oncol.

[35] Corso CD, Rutter CE, Wilson LD, Kim AW, Decker RH, Husain ZA (2015). Re-evaluation of the role of postoperative radiotherapy and the impact of radiation dose for non-small-cell lung cancer using the National Cancer Database. J Thorac Oncol.

[36] Wang HH, Deng L, Wen QL, Zhang CZ, Zaorsky NG, Zhang BL (2017). Early postoperative radiotherapy is associated with improved outcomes over late postoperative radiotherapy in the management of completely resected (R0) stage IIIA-N2 non-small cell lung cancer. Oncotarget.

[37] Lee HW, Noh OK, Oh YT, Choi JH, Chun M, Kim HI (2016). Radiation therapy-first strategy after surgery with or without adjuvant chemotherapy in stage IIIA-N2 non-small cell lung Cancer. Int J Radiat Oncol Biol Phys.

[38] Booth CM, Shepherd FA, Peng Y, Darling G, Li G, Kong W (2013). Time to adjuvant chemotherapy and survival in non-small cell lung cancer: a population-based study. Cancer.

[39] Singh AK, Winslow TB, Kermany MH, Goritz V, Heit L, Miller A (2017). A pilot study of stereotactic body radiation therapy combined with Cytoreductive nephrectomy for metastatic renal cell carcinoma. Clin Cancer Res.

